# Chondroitin Sulfate Prevents STZ Induced Diabetic Osteoporosis through Decreasing Blood Glucose, AntiOxidative Stress, Anti-Inflammation and OPG/RANKL Expression Regulation

**DOI:** 10.3390/ijms21155303

**Published:** 2020-07-26

**Authors:** Hong Xing Zheng, De Jing Chen, Yue Xin Zu, En Zhu Wang, Shan Shan Qi

**Affiliations:** 1 College of Biological Science and Engineering, Shaanxi University of Technology, Hanzhong 723000, China; cdjslg@126.com (D.J.C.); jjlin1306@163.com (Y.X.Z.); a371505153@163.com (E.Z.W.); 2 Shaanxi Province Key Laboratory of Bio-Resources, Shaanxi University of Technology, Hanzhong 723000, China

**Keywords:** diabetic osteoporosis, chondroitin sulfate, oxidative stress, bone-mineral density

## Abstract

Chondroitin sulfate (CS) has antioxidative, anti-inflammatory, anti-osteoarthritic and hypoglycemic effects. However, whether it has antidiabetic osteoporosis effects has not been reported. Therefore, in this study, we established a STZ-induced diabetic rat model; CS (500 mg kg^−1^ d^−1^) was orally administrated for eight weeks to study its preventive effects on diabetic osteoporosis. The results showed that eight weeks of CS treatment improved the symptoms of diabetes; the CS-treated group has increased body weight, decreased water or food intake, decreased blood glucose, increased bone-mineral density, repaired bone morphology and decreased femoral osteoclasts and tibia adipocytes numbers. After CS treatment, bone histomorphometric parameters returned to normal, the levels of serum inflammatory cytokines (IL-1β, IL-6 and TNF-α) decreased significantly, serum SOD, GPX and CAT activities increased and MDA level increased. In the CS-treated group, the levels of serum ALP, CTX-1, TRACP 5b, osteocalcin and RANKL decreased and the serum RUNX 2 and OPG levels increased. Bone immunohistochemistry results showed that CS can effectively increase the expression of OPG and RUNX2 and reduce the expression of RANKL in diabetic rats. All of these indicate that CS could prevent STZ induced diabetic osteoporosis—mainly through decreasing blood glucose, antioxidative stress, anti-inflammation and regulation of OPG/RANKL expression. CS can therefore effectively prevent bone loss caused by diabetes.

## 1. Introduction

With the aging of the population and unhealthy diets, the incidence of diabetes is increasing year-by-year, and has become the third largest chronic noncommunicable disease following tumors and cardiovascular diseases, seriously affecting the health of patients [1]. Diabetic osteoporosis (DOP) is a systemic, metabolic bone disease that seriously affects the health and quality of life of patients. Among people with diabetes, the longer the duration of diabetes, the higher the incidence of osteoporosis, which can be as high as 50% to 60% [2]. The main pathologic features of DOP are decreased bone mass per unit volume, altered bone tissue microstructure, decreased bone strength, increased brittleness and increased risk of fractures [3,4].

Studies have shown that the pathogenesis of DOP may be closely related to hyperglycemia, oxidative stress and inflammatory mediators [5,6,7,8]. Hyperglycemia stimulating the body continuously produce and accumulate advanced glycation end products (AGEs) in diabetic patients. The high levels of AGEs in the body can affect the bone metabolism proteins expression and bone cell differentiation, resulting in reduced bone formation and reduced bone density, thus leading to osteoporosis [9]. There also exist oxidative stress in the body of diabetic patients, and the level of active oxygen increased in diabetes. Studies have shown that active oxygen is involved in regulating the formation and activity of osteoblasts and osteoclasts and is involved in the occurrence and development of osteoporosis [10]. In addition, inflammatory cytokines and homocysteine are playing important roles in the development of DOP, they can increase osteoclastogenesis, leading to increased bone resorption and osteoporosis [11,12,13,14].

Chondroitin sulfate (CS) is a kind of sulfated glycosaminoglycan and is an acidic mucopolysaccharide widely expressed in animal connective tissues [15]. CS has antioxidant, anti-inflammatory, anti-thrombotic and antiosteoarthritic effects, among others [16,17,18]. Recent studies have found that it has a hypoglycemic and antidiabetic effects and could improve insulin sensitivity of diabetes [19,20,21]. However, whether it has anti-DOP effect has not been reported. Therefore, in this study, we established an STZ-induced diabetic model to study the preventive effect of CS on DOP.

## 2. Results

### 2.1. CS Increased Body Weight and Decreased Water and Food Intake

**Figure 1:**
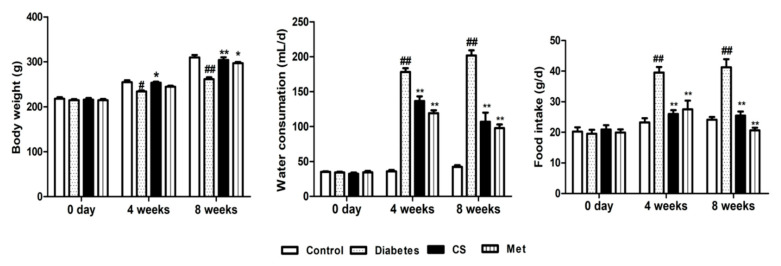
Body weight, water and food intake of rats in each group. ^##^ *p* < 0.01 compared with the control group, ^#^ *p* < 0.05 compared with the control group; ** *p* < 0.01 compared with the diabetic group, * *p* < 0.05 compared with the diabetic group.

As Figure 1 shows, there were no differences of the levels of body weight, food or water intake between groups at the initial of study. Body weight was reduced in the diabetic group at the 4th and 8th week of study (diabetes vs. control, *p* < 0.05); the food or water intake was significantly increased in diabetic group at the 4th and 8th week of the study (diabetes vs. control, *p* < 0.01). After CS administration the body weight of CS group increased at the 4th week of the study (CS vs. diabetes, *p* < 0.05), at the same time, the water or food intake decreased in CS group and Met group (CS vs. diabetes, *p* < 0.01, Met vs. diabetes, *p* < 0.01). Suggesting that CS could improve the signs of diabetes.

### 2.2. CS Increased Bone-Mineral Density (BMD) of Diabetic Rats

**Figure 2:**
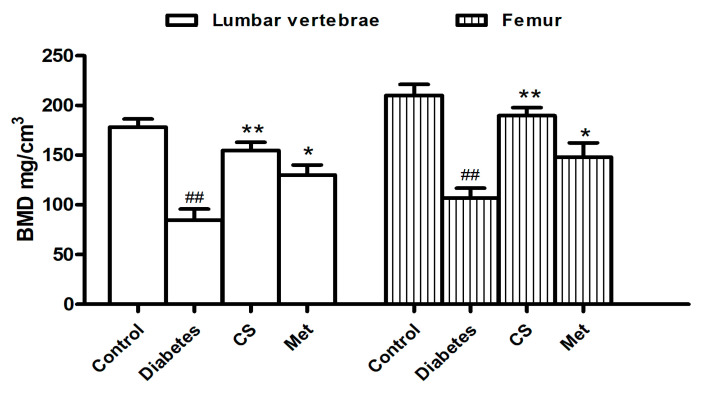
Lumbar vertebrae and femur bone-mineral density (BMD) of each group at the end day of the animal experiment. ^##^ *p* < 0.01 compared with the control group; ** *p* < 0.01 compared with the diabetic group, * *p* < 0.05 compared with the diabetic group.

As shown in Figure 2, both femur and lumbar spine BMD of diabetic rats significantly decreased (diabetes vs. control, *p* < 0.01); however, after eight week chondroitin sulfate (CS) or metformin (Met) administration, BMD significantly increased compared with the diabetic group (*p* < 0.01 and *p* < 0.05, respectively).

### 2.3. CS Decreased Blood Glucose and Regulated Serum Bone Turn over Markers

As Table 1 shows, blood glucose, as well as the levels of ALP, CTX-1, TRACP 5b, osteocalcin, PINP, and RANKL in serum increased in the diabetic group (diabetes vs. control, *p* < 0.01) and the RUNX 2—as well as OPG levels decreased (diabetes vs. control, *p* < 0.01). Eight weeks CS or metformin administration decreased the levels of ALP, CTX-1, TRACP 5b, osteocalcin, PINP and RANKL (CS vs. diabetes, *p* < 0.01), increased the levels of RUNX 2 and OPG (CS vs. diabetes, *p* < 0.01, Met vs. diabetes, *p* < 0.05). The OPG/RANKL ratio increased after CS treatment (CS vs. diabetes, *p* < 0.01). These findings suggest that CS could regulate serum bone turn over markers.

### 2.4. CS Decreased Serum Inflammatory Cytokines

**Figure 3:**
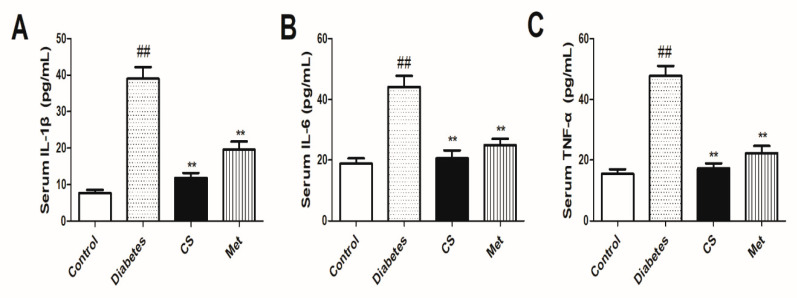
Serum levels of serum inflammatory cytokines (IL-1β, IL-6) and tumor necrosis factor-α (TNF-α) in each group at the end day of the animal experiment. (**A**) serum IL-1β levels in each group; (**B**) serum IL-6 levels in each group; (**C**) serum TNF-α levels in each group. ^##^ *p* < 0.01 compared with the control group; ** *p* < 0.01 compared with the diabetic group.

As Figure 3 shows, at the end day of the animal experiment, the serum levels of IL-1β, IL-6, and TNF-α in diabetic group increased significantly (diabetes vs. control, *p* < 0.01). Eight weeks CS or metformin administration decreased IL-1β, IL-6 and TNF-α levels (CS vs. diabetes, *p* < 0.01, Met vs. diabetes, *p* < 0.01). All of these indicated that CS could inhibit serum inflammatory cytokines in diabetic rats.

### 2.5. CS Inhibited Oxidative Stress of Diabetic Rats

**Figure 4:**
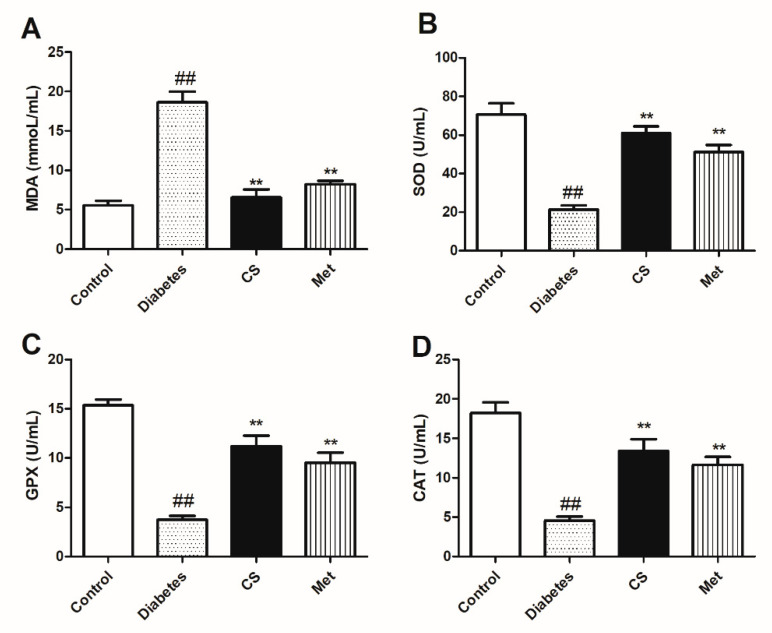
Serum levels of MDA concentration, superoxide dismutase (SOD), glutathione peroxidase (GPX) and catalase (CAT) activities in each group at the end day of the animal experiment. (**A**) serum MDA level in each group; (**B**) serum SOD activity in each group; (**C**) serum GPX activity in each group; (**D**) serum CAT activity in each group. ^##^ *p* < 0.01 compared with the control group; ** *p* < 0.01 compared with the diabetic group.

As Figure 4 shows, at the end day of the study, there were increased MDA levels (diabetes vs. control, *p* < 0.01), decreased SOD, GPX and CAT activities (diabetes vs. control, *p* < 0.01) in diabetic group, after CS or metformin administration the levels of MAD decreased (CS vs. diabetes, *p* < 0.01, Met vs. diabetes, *p* < 0.01)*,* the SOD, GPX, and CAT activities increased (CS vs. diabetes, *p* < 0.01, Met vs. diabetes, *p* < 0.01). These indicated that CS has antioxidative stress function in diabetic rats.

### 2.6. CS Repaired Bone Tissue Structure in Diabetic Rats

**Figure 5:**
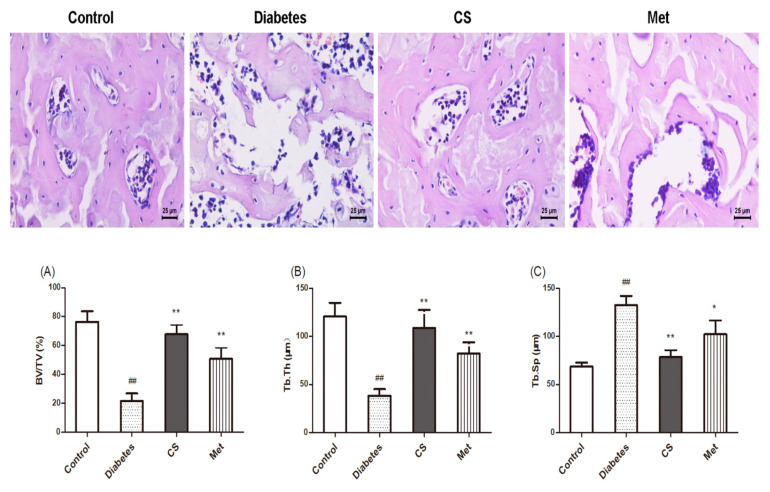
Morphology of femoral bone of rats in each group, hematoxylin and eosin staining, magnification, 400×. (**A**) Percentage of trabecular volume in each groups; (**B**) trabecular thickness in each groups; (**C**) trabecular spacing in each groups. ^##^ *p* < 0.01 compared with the control group; ** *p* < 0.01 compared with the diabetic group, * *p* < 0.05 compared with the diabetic group.

As Figure 5 shows, there were fractured and sparseness trabecular in femur of diabetic rats. Bone histomorphometric parameters (Figure 5A–C) indicated that the percentage of trabecular volume and trabecular thickness in femur of diabetic rats decreased (diabetes vs. control, *p* < 0.01), whereas they increased in CS or Met-treated groups (CS vs. diabetes, *p* < 0.01, Met vs. diabetes, *p* < 0.01). The increased trabecular spacing observed in diabetic group (diabetes vs. control, *p* < 0.01) was repaired by CS treatment (CS vs. diabetes, *p* < 0.01).

### 2.7. CS Inhibits Osteoclastogenesis in Diabetic Rats

**Figure 6:**
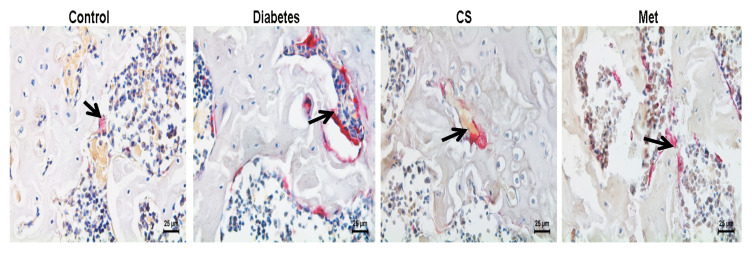

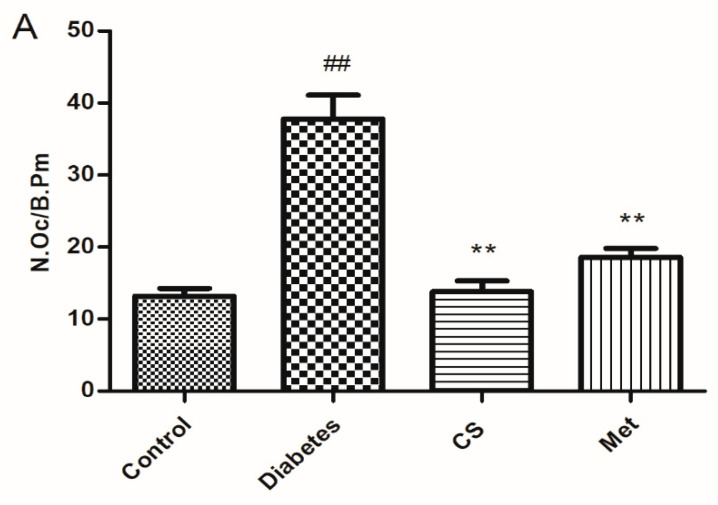
(**A**) Graph illustrates the number of osteoclasts per bone perimeter in each group at the end day of experiment (N.Oc/B.Pm.), TRAP-staining, magnification, 400×. Count chart of the number of osteoclasts in each group at the end day of experiment. N.Oc/B.Pm. number of osteoclasts per bone perimeter. ^##^ *p* < 0.01 compared with the control group; ** *p* < 0.01 compared with the diabetic group. The black arrows points to osteoclasts.

As shown in Figure 6, osteoclasts were red after TRAP staining, distributed around the trabecular bone. As Figure 6A shows, the number of femoral osteoclasts increased in diabetic group (diabetes vs. control, *p* < 0.01); eight weeks of CS or metformin administration decreased the femoral osteoclasts numbers significantly (CS vs. diabetes, *p* < 0.01, Met vs. diabetes, *p* < 0.01).

### 2.8. CS Inhibits Bone Marrow Adipocytes in Diabetic Rats

**Figure 7:**
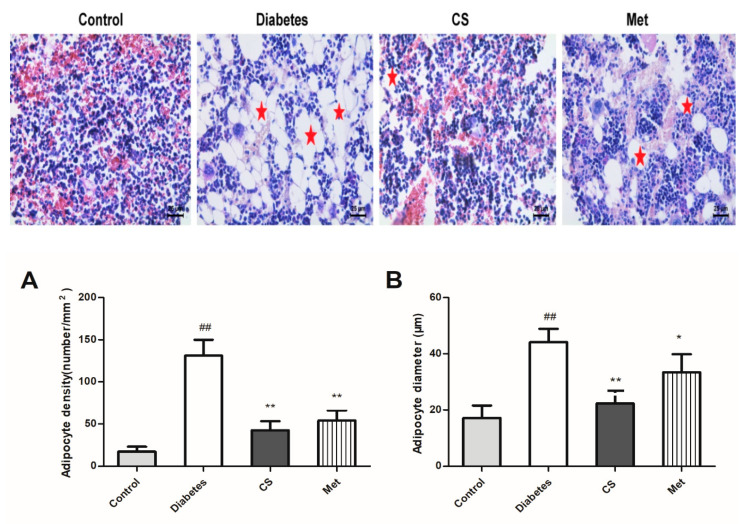
Adipocytes in the bone marrow cavity of the tibia, hematoxylin and eosin staining, magnification, 400×. (**A**) Adipocytes density in each group at the end day of experiment; (**B**) adipocytes diameter in each group at the end day of experiment. ^##^ *p* < 0.01 compared with the control group; ** *p* < 0.01 compared with the diabetic group, * *p* < 0.05 compared with the diabetic group. The red stars in figures showing adipocytes.

As Figure 7 indicated, large amount of adipocytes is distributed in the bone marrow cavity of the tibia of diabetic rats. As shown in Figure 7A,B, adipocytes density and diameter increased in diabetic rats tibia (diabetes vs. control, *p* < 0.01), whereas they decreased after CS or metformin treatment (adipocytes density, CS vs. diabetes, *p* < 0.01, Met vs. diabetes, *p* < 0.01; adipocytes diameter, CS vs. diabetes, *p* < 0.01, Met vs. diabetes, *p* < 0.05).

### 2.9. CS Regulated Bone OPG, RANKL and RUNX 2 in Diabetic Rats

**Figure 8:**
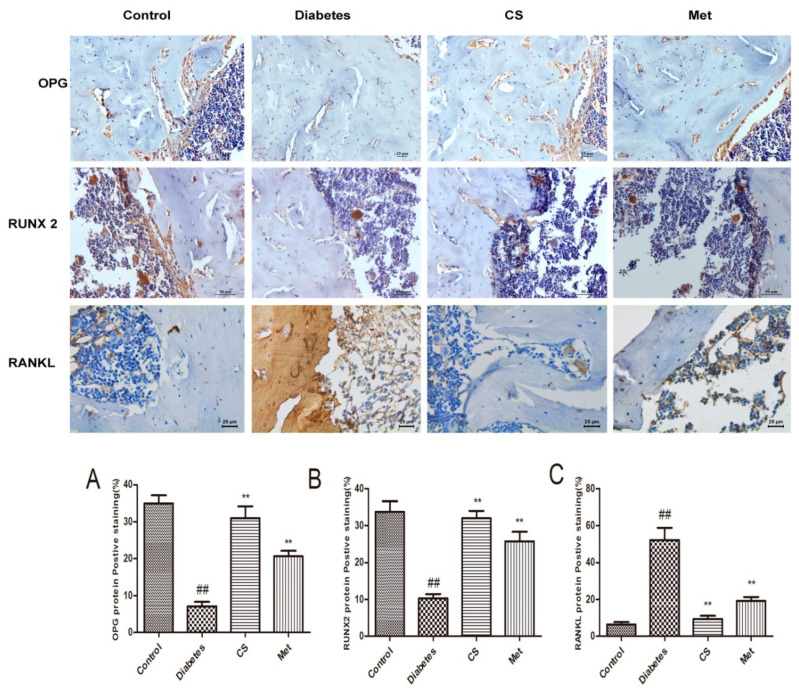
Proteins (OPG, RUNX 2 and RANKL) in the femurs of each group rats at the end day of experiment, immunohistochemical staining, magnification, 400×. (**A**) Positive-staining area of OPG in each group; (**B**) positive-staining area of RUNX 2 in each group; (**C**) positive-staining area of RANKL in each group. ^##^ *p* < 0.01 compared with the control group; ** *p* < 0.01 compared with the diabetic group.

As Figure 8 shows, the OPG and RUNX 2 positive-staining area decreased (diabetes vs. control, *p* < 0.01), whereas RANKL positive-staining area increased (diabetes vs. control, *p* < 0.01) in diabetic rats femur. The OPG and RUNX 2 positive-staining area increased (CS vs. diabetes, *p* < 0.01, Met vs. diabetes, *p* < 0.01) and the RANKL positive-staining area decreased after CS or metformin treatment (CS vs. diabetes, *p* < 0.01, Met vs. diabetes, *p* < 0.01). These findings indicated that CS could increase the expression of OPG and RUNX 2 and inhibit the bone RANKL expression in the bone of diabetic rats.

## 3. Discussion

Among people with diabetes, the longer the duration of diabetes, the higher the incidence of osteoporosis, which can be as high as 50% to 60% [2]. In the present study, we found that the BMD of diabetic rats decreased, whereas, giving CS treatment for eight weeks, the BMD increased significantly, the bone morphology was repaired, femoral osteoclasts number decreased and the bone histomorphometric parameters turned out to be normal. Another important finding was that CS has a good hypoglycemic effect and could relieve many signs of diabetes (such as weight loss, eating more, drinking more) and this was consistent with the study of Motoab et al. [19].

The most important feature of diabetes is elevated blood glucose, and hyperglycemia is closely related to osteoporosis. It has been shown that long-term hyperglycemia can cause osmotic diuresis, promote the excretion of calcium, phosphorus, magnesium and other electrolytes, resulting in decreased bone-mineral density [22,23]; Moreover, hyperglycemia can cause excessive accumulation of AGEs in the body; AGEs bind to the surface receptors on monocytes and macrophages, inducing them to produce various inflammatory factors, such as interleukin (IL)-1, IL-6, tumor necrosis factor-α (TNF-α), etc. These cytokines accelerate the conversion of osteoclast precursors into mature osteoclasts, promote osteoclast activity and accelerate bone resorption process, leading to bone loss [24]. AGE can also act as an alarmin in diabetes and osteoporosis. The receptor for advanced glycation end-products (RAGE) is involved in bone remodeling, and CS could also directly counteract the AGE/RAGE axis induced osteoporosis in diabetes as well as in other pathologies leading to osteoporosis [25]. In the present study, the blood glucose level of diabetic rats decreased after CS treatment, and the levels of serum inflammatory cytokines (IL-1β, IL-6 and TNF-α) were also decreased significantly. Hence, we guess the reducing blood glucose was one of the important reasons for CS to exert anti-osteoporotic effects in diabetes.

Patients with diabetes are often accompanied by chronic metabolic inflammation, IL-1β, IL-6 and TNF-α and other inflammatory cytokines are upregulated [26]. Inflammatory cytokines can inhibit osteoblasts differentiation by enhancing osteoclasts activity, leading to osteoporosis. TNF-α can increase RANKL expression, inhibit collagen synthesis, ALP activity and osteocalcin synthesis and promote bone loss [27]. IL-6 can stimulate osteoclasts precursor cell division and proliferation, enhance its activity and promote osteoclasts differentiation [28]. IL-1β can stimulate osteoclasts to produce RANKL, thereby promoting bone resorption and causing bone loss [29]. In this study, CS treatment of diabetic rats increased BMD and decreased inflammatory cytokines, osteoclast number and RANKL expression, suggesting that CS exerts an anti-osteoporotic effect in diabetes also through its anti-inflammatory function.

Oxidative stress (OS) is one of the important mechanisms leading to DOP, studies suggested that drugs with antioxidant effects may have a protective effect on diabetes-induced bone loss [30]. Under physiological conditions, the generation and removal of active oxygen in the body are in a state of dynamic equilibrium, so they have no harmful effects on the body. In diabetes, increased blood glucose significantly increases the level of AGEs in the blood circulation and tissues and AGEs bind to their receptors (RAGE) thus increased ROS production in tissues, ROS can induce osteoblasts and bone marrow stromal cells to express RANKL, promote osteoclasts formation, differentiation and maturation, thus increasing bone resorption, and ultimately leading to bone tissue damage and DOP [31]. MDA is the final product of lipid oxidation, so the amount of MDA can reflect the degree of lipid peroxidation in the body, which indirectly reflects the degree of cell damage. SOD, GPX and CAT were important antioxidant enzyme systems in the body, their activities indirectly reflect the body’s ability to resist OS. In this study, MAD level decreased, SOD, GPX and CAT activities increased after CS treatment, indicated that CS has antioxidative stress ability, and it was one of reasons that CS exerts antidiabetic osteoporosis effect.

Bone turnover biomarkers refer to a class of biomolecules that are produced during bone remodeling and can be measured in urine or serum. It could be divided into bone formation markers and bone resorption markers [32]. ALP, PINP and osteocalcin are bone formation markers representing the metabolic products of osteoblasts activity and bone formation; CTX-1 and TRACP 5b are bone resorption markers, representing the metabolites of osteoclasts activity and bone resorption, especially the degradation products of bone matrix [33]. In this study, both bone formation and bone resorption markers increased in diabetic rats, indicating an accelerated bone turnover in diabetes. The bone formation markers (ALP, PINP and osteocalcin) and bone resorption markers (CTX-1 and TRACP 5b) were all decreased after CS treatment suggesting that CS could decrease the bone turnover in diabetic rats.

OPG, also known as osteoprotegerin, is a member of tumor necrosis factor family and a protective factor for bone metabolism, which function is to compete with RANK to link RANKL thus decreasing osteoclastogenesis, reducing osteoclast activity and hindering bone resorption [34]. At the same time, OPG can promote osteoclasts apoptosis [35]. The decreased OPG expression observed in diabetes could represent an additional pathogenetic factor for osteoporosis [36]. Many studies show that the OPG/RANKL system has an important impact on the pathogenesis of osteoporosis; the decreased OPG/RANKL ratio and the relative increase of RANKL ligands, promote osteoclast differentiation and maturation, thereby promoting bone resorption. When the ratio of OPG/RANKL is increased, the ligand of RANKL is relatively reduced, which can reduce the activation of osteoclasts, thereby inhibiting bone resorption and increasing bone formation function [37]. In summary, OPG/RANKL system have a very close relationship with osteoporosis. In the present study, serum and bone OPG expression levels were upregulated and RANKL levels were downregulated by CS, meanwhile, the serum OPG/RANKL ratio was upregulated by CS, which indicated reduced osteoclasts activity and increased bone formation function.

RUNX2 is a bone specific transcription factor. It is an important sign of osteoblasts differentiation and the process of bone formation [38]. High expression of RUNX2 can activate the transcription of bone sialoprotein (BSP) and osteocalcin, thereby promoting the maturation of osteoblasts and bone formation [39]. Epidemiological investigation found that the expression of RUNX2 in diabetic osteoporosis patients was significantly reduced [40], which is consistent with the results of this study. In this study, the decreased levels of RUNX 2 in serum and bone tissues of diabetic rats were upregulated by CS, indicating that CS could increase bone formation function of diabetic rats.

Bone marrow adipocytes, and osteoblasts are mainly differentiated from bone marrow mesenchymal stem cells (MSCs), and bone marrow adipocytes and osteoblasts have a competitive relationship [41]. During osteoporosis (including diabetic osteoporosis), the number and activity of bone marrow osteoblasts decreased and adipocytes increased. The increased number of bone marrow adipocytes were closely associated with a decreased BMD [42]. Clinical and histopathologic studies also confirm the above statement [43]. In our present study, the number of tibia bone marrow adipocytes—which increased in diabetic rats—decreased following CS treatment, suggesting that CS could inhibit MSCs differentiating to adipocytes.

Metformin is a classic hypoglycemic drug. In vivo and in vitro studies have reported that it could promote osteoblasts differentiation and increase bone-mineral density in diabetes [44,45,46]. We used metformin as a positive control in this study and also found similar efficacy. In this study, it was found that although the hypoglycemic effect of metformin was better than CS, the anti-DOP effect of CS was superior to that of metformin. We only revealed the anti-DOP effect of CS in terms of antioxidant, anti-inflammatory, regulatory capacity on bone turnover and OPG/RANKL system, etc. and the animal number used in this study was not so large enough. In this study, we have not designed a group of CS treatment group in healthy rats, if this group was designed, it will be better to confirm its antioxidation effects. However, many studies have revealed the antioxidation effects of CS [47,48]. Due to the complex pathogenesis of DOP, the molecular mechanism of CS against DOP needs further studies. Besides DOP, it was also reported CS has the potential clinical use among other emerging osteoporosis therapies targeted to immunological checkpoints [49].

## 4. Materials and Methods

### 4.1. Animals and Reagents

Seven-week-old female SD rats (weight 205–220 g) were purchased from Chengdu Dashuo Experimental Animal Company. The rats were housed in the standardized animal room of Shaanxi University of Technology (temperature 20–23 °C, humidity 45%–50%). The animal experiment was approved by the Experimental Animal Ethics Committee of Shaanxi University of Technology (Approval No. 2019-037, Approval Date 15 July 2019). Chondroitin sulfate used in this study was from bovine trachea (C 9819, Sigma, St. Louis, MO, USA).

### 4.2. Diabetes Model Induction and Treatment

Rats were randomly divided into two groups (a control group and a model group) after seven days of adaptive feeding. The model group rats were intraperitoneally injected with streptozotocin (45 mg/kg, dissolved in a citrate buffer solution pH 4.5). The control group rats were injected with an equal volume of citrate buffer solution. Seventy-two hours after injection, random blood glucose was detected. Rats with random blood glucose ≥ 11.1 mmol/L were regarded as diabetic rats.

After the diabetic model was constructed, the animals were divided into four groups, namely, the control group, diabetes group, CS group and Met group. There were 10 rats in each group. The CS group was given 500 mg kg^−1^ d^−1^ chondroitin sulfate (dissolved in distilled water) by gavage for 8 weeks; The Met group was given 200 mg kg^−1^ d^−1^ metformin (dissolved in distilled water) by gavage for 8 weeks; The control group and model group were given distilled water every day. During the experiment, blood glucose, body mass, water intake and food intake were monitored.

### 4.3. Bone-Mineral Density Measurement

On the last day of the experiment, the bone-mineral density of the femur and vertebrae of each group of rats was measured using a dual-energy X-ray bone densitometer (RZ-Digumus, Kubtec, Peoria, IL, USA).

### 4.4. Serum Inflammatory Cytokines Detection

On the last day of the animal study, rats were anesthetized with isoflurane and blood was collected from the orbital veins. Serum was collected after centrifugation and stored in a refrigerator at −80 °C until use. The level of serum TNF-α, IL-1β and IL-6 were measured by Elisa kits (Sino Biologic, Beijing, China) according to the protocols listed in the kits.

### 4.5. Serum Bone Turnover Marker Detection

The level of serum ALP, CTX-1, TRACP 5b, osteocalcin, RANKL and RUNX 2 were measured by Elisa kits (Sino Biologic, Beijing, China) according to the protocols listed in the kits.

### 4.6. Serum Oxidative Stress Index Detection

Serum SOD activity, MDA content, CAT activity and GSH-Px activity were determined by commercial kit (Sino Biologic, Beijing, China) according to the protocols listed in the kits.

### 4.7. Bone Tissue Morphology and Bone Morphometric Parameters

The left femoral tissues were placed in 10% EDTA solution for 28 days. After dehydration with ethanol gradient, the bone tissue was placed in 80% ethanol for 1 h, 95% ethanol overnight and 100% ethanol for 1 h. Then, the bone tissues were placed in xylene solution for 15 min and placed in paraffin for 3 h. Then, the bone tissue was embedded in paraffin and sliced to prepare bone tissue slices with a thickness of 5 μm using a paraffin tissue slicer (Leica, RM2235, Dublin, Ireland). The bone tissue slices were stained with hematoxylin and eosin staining solution.

Femoral tissue morphology observation and image acquisition were performed using a Leica microphotography system. Then we used Image-Pro Plus 5.0 analysis software to perform morphologic measurements. According to the correction formula, the percentage of trabecular volume, trabecular thickness (Tb. Th), trabecular spacing (Tb. Sp) in each groups were calculated [50].

### 4.8. Tibia Bone Marrow Adipocytes Observation

We randomly selected five non-overlapping fields of view at 400× in each hematoxylin and eosin stained tibia tissue samples and counted all bone marrow adipocytes in each field of view. The average value of each field of view was taken as the bone marrow adipocytes count value.

### 4.9. Bone TRAP Staining for Osteoclasts Observation

Rat femoral tissue sections were put in xylene solution for 8 min, and then put it in 100% ethanol, 95% ethanol, 80% ethanol (5 min each), the they were washed in deionized water for 3 min, stained with TRAP staining solution (pH 5.0–5.2) at 37 °C in the dark for 3 h, then the slides were washed thoroughly with distilled water and stained with hematoxylin for 1 min, after then, washed the slides with distilled water, then the slices are gradually dehydrated by gradient ethanol (80%, 95% I, 95% II, 100% I, 100% II). Then observe the morphology of femoral osteoclasts under an optical microscope. Randomly select five non-overlapping fields of view at 400× in each slide, the number of osteoclasts in each field of vision was accurately counted.

### 4.10. Bone OPG, RANKL, RUNX 2 Immunohistochemical Staining

Five-micrometer-thick rat femoral paraffin tissue sections were soaked in xylene three times for 10 min each time; sections were put in gradient ethanol (100% I, 100% II, 95%, 80%, 50%) for 5 min each; Sections were washed thoroughly in PBS-T and then placed in 1% TritonX-100 solution and soak for 30 min. Sections were then placed in 90 °C citrate buffer for 20 min and soaked in 3% H2O2 solution for 30 min. Sections were washed thoroughly with PBS; 3% BSA solution was added, incubated in the room temperature for 20 min and washed thoroughly in PBS-T. The primary antibodies (OPG, RANKL, RUNX 2) were diluted at a ratio of 1:100 and added on the respective slices. After 2 h incubation at 37 °C, slices were thoroughly washed with PBS-T and the secondary antibody (HRP labeled goat ant-rabbit IgG), diluted at a ratio of 1:250, was added on each slice. Following 1 h incubation at 37 °C, slices were washed thoroughly with PBS-T. DAB incubation for 1 min, and hematoxylin counter-stain for 1 min were performed. Slices were put in graded ethanol (80%, 95% I, 95% II, 100% I, 100% II) and then the stained sections were observed under the light microscope. After randomized selection different fields of view, pictures were taken and saved. Finally, Image Pro Plus 5.0 analysis software was used to quantitatively analyze the percentage of the OPG, RANKL, RUNX 2 positive stained area (including matrix area).

### 4.11. Statistical Analysis

Data calculation results are expressed as mean ± SEM. The significance of the difference between groups was determined by analysis of variance (ANOVA) followed by Tukey’s test, *p* < 0.05 was considered significant.

## 5. Conclusions

Chondroitin sulfate has hypoglycemic effect and could prevent STZ induced diabetic osteoporosis, increase the BMD and repair bone structure of diabetic rats. The possible mechanisms of its anti-DOP effects could be exerted mainly through decreasing blood glucose, antioxidative properties, anti-inflammation and modulating OPG/RANKL expression. This study will provide basic data for the application of chondroitin sulfate in the treatment of DOP.

## Figures and Tables

**Table 1:** Blood glucose and serum bone turnover markers in each group of rats at the end of the experiment.

Parameter	Control	Diabetes	CS	Met
Blood glucose (mg dL^−1^)	91.76 ± 6.94	401.85 ± 21.41 ^##^	302.56 ± 16.77 **	226.74 ± 15.79 **
ALP (U dL^−1^)	89.78 ± 9.56	197.54 ± 11.70 ^##^	99.89 ± 912 **	143.51 ± 7.34 *
CTX-1 (ng mL^−1^)	31.20 ± 4.57	126.14 ± 13.32 ^##^	46.89 ± 6.01 **	74.31 ± 7.68 **
Osteocalcin (ng mL^−1^)	16.78 ± 3.41	52.42 ± 5.31 ^##^	20.46 ± 3.59 **	29.41 ± 6.48 **
TRACP 5b (U dL^−1^)	2.41 ± 0.44	5.37 ± 1.20 ^##^	2.87 ± 0.59 **	3.96 ± 1.13 *
PINP (μg L^−1^)	29.67 ± 3.95	69.80 ± 8.47 ^##^	33.59 ± 3.90 **	51.78 ± 6.51 *
RUNX 2 (ng mL^−1^)	13.10 ± 3.18	3.64 ± 0.57 ^##^	10.89 ± 2.91 **	6.48 ± 2.10 *
OPG (ng mL^−1^)	8.90 ± 1.48	2.39 ± 0.69 ^##^	7.54 ± 0.94 **	4.89 ± 1.04 *
RANKL (ng mL^−1^)	2.01 ± 0.38	9.45 ± 1.76 ^##^	3.38 ± 0.57 **	5.80 ± 1.67 *
OPG/RANKL ratio	4.36 ± 0.41	0.27 ± 0.08 ^##^	2.51 ± 0.33 **	0.89 ± 0.04 *

^##^*p* < 0.01 compared with the control group; ** *p* < 0.01 compared with the diabetic group, * *p* < 0.05 compared with the diabetic group.

## Abbreviations

DOP	Diabetic osteoporosis
CS	Chondroitin sulfate
OPG	Osteoprotegerin
RANKL	Receptor activator of nuclear factor-κ B ligand
RUNX2	Runt-related transcription factor 2
STZ	Streptozotocin
CAT	Catalase
SOD	Superoxide dismutase
MDA	Malondialdehyde
GSH-Px	Glutathione peroxidase
AGEs	Advanced glycation end products
OS	Oxidative stress
CTX-1	Cross-linked C-telopeptide of type I collagen
P1NP	Procollagen I N-terminal propeptide
BMSCs	Bone marrow stromal cells
ALP	Alkaline phosphatase
TRACP 5b	Tartrate-resistant acid phosphatase 5b
